# A Holistic Review of 3-Dimethylamino-1-Arylpropenones Based Disperse Dyes for Dyeing Polyester Fabrics: Synthesis, Characterization, and Antimicrobial Activities

**DOI:** 10.3390/polym16040453

**Published:** 2024-02-06

**Authors:** Alya M. Al-Etaibi, Morsy Ahmed El-Apasery

**Affiliations:** 1 Natural Science Department, College of Health Science, Public Authority for Applied Education and Training, Fayha 72853, Kuwait; 2 Dyeing, Printing and Textile Auxiliaries Department, Textile Research and Technology Institute, National Research Centre, 33 El Buhouth St., Dokki, Cairo 12622, Egypt; elapaserym@yahoo.com

**Keywords:** disperse dyes, polyester fabrics, self-cleaning, UV protection, ZnO NPs

## Abstract

The enaminone compounds 3-Dimethylamino-1-arylpropenones produced in this review was synthesized by reacting *para*-methylacetophenone and *para*-nitroacetophenone with dimethylformamide dimethyl acetal. In this review article, we discuss how to create novel disperse colors by reacting enaminone derivatives **3a** and **3b** with phenyldiazonium salt. The highly productive procedure of creating new disperse dyes was followed by the process of dyeing polyester fabrics at temperatures between 70 and 130 °C. As a result, the colours’ resistance to light, rubbing, perspiration, and washing fastness was assessed. In an effort to show the additional value of these dyes, the expected biological activity of the synthetic dyes against fungus, yeast, and Gram-positive and Gram-negative bacteria was also assessed. We have applied zinc oxide nanoparticles for polyester fabrics treatment to impact them a self-cleaning quality, increase their light fastness, enhance their antibacterial efficacy, and enhance UV protection as part of our ongoing strategy to obtain polyester fabrics with newly acquired specifications.

## 1. Introduction

Dyes have played an important and pivotal role since the beginning of human civilization. For a very long time, these sources remained the only means of producing colors. Natural dyes were made from natural sources, such as minerals, plants, insects, and animals. Bright colors ranging from black to yellow were present in these sources [1,2,3,4]. The use of artificial dyes with natural dyes started as civilization advanced and demand rose. The range of colors that are visible to the human eye is 400–700 nm [5,6,7]. Synthetic dyes are widely used to add color to a variety of products, including paper, plastics, leather, and textiles [8,9,10,11]. But their applications are not limited to these sectors; the food, pharmaceutical, and cosmetics industries also make extensive use of them. These synthetic dyes are produced using both organic and inorganic compounds that absorb light between 400 and 800 nm in the visible spectrum. The molecules contain different chromophores present in the pigment, which leads to the appearance of a very large number of colors. There is also a tinting process that takes place between the colors to obtain infinite numbers of bright colors, and this is what distinguishes these dyes [12,13,14,15,16,17]. The structure or components of the dyes are essentially what determine how much light is absorbed. Because dyes are easily obtained in the numbers and standards needed in each sector, this forms the basis for their application in a variety of fields and sectors. Azo dyes represent the largest and most diverse chemical class of all organic dyes. Approximately 75% of all organic dyes produced fall into this category [18,19,20,21,22]. One increasingly flexible class of organic dyes is azo dyes. The excellent cumulative profile, high dye strength, brighter shades, and superior stability properties of disperse azo dyes based on enaminones, along with their intriguing biological properties and diverse applications; have drawn attention in the field of disperse dye chemistry. It is well known that the first production of polyester (PET) was achieved in 1941. Polyester production has revolutionized the textile industry [23,24,25,26,27,28,29,30,31,32,33,34,35,36,37,38,39,40,41,42]. Polyester fibers are defined as long-chain polymers composed of dihydric alcohol ester and terephthalic acid [23,24]. Because PET has better qualities than other polyester fibres, it is the most widely used type of fibres [43]. Since polyester is produced at a higher rate than both polyamide and polyacrylic combined, it is the synthetic fabrics that are utilized the most extensively. Good tensile strength, high chemical resistance, high resistance to microorganisms like bacteria, light weight, relatively low cost, corrosion resistance, stability against sunlight, acid reduction and oxidation, and stability against most organic solvents are some of the qualities that set polyester apart. Because PET fibres are hydrophobic by nature and have a glass transition temperature (Tg) of roughly 80 °C, the dyeing process is impacted and becomes more challenging because it requires a lot of energy and water [25,26,27]. Disperse dyes are the most often used dyes for coloring PET fabrics. Since these dyes are nearly insoluble in water, it is required to use disperse dyes with carriers and dispersing agents if the dyeing processes are carried out in water that is below/equals 100 °C or to use disperse dyes with dispersing agents if the water temperature is 130 °C. With the aid of carriers, the dyeing process is carried out at low temperatures below 100 °C and at normal atmospheric pressure. Carriers contribute to the disperse colors’ increased solubility in water. The process of dispersing dyes into polyester and absorbing carriers into polyester fabric are remarkably similar, with the aromatic components of the carriers adhering to the polyester fibres [28,29,30,31,32,33]. The molecular weight of the carriers is less than that of the distributed dyes when compared. The carriers increase the absorption of the disperse dye at temperatures higher than the glass transition temperature (Tg) of polyester by penetrating into the amorphous portions of the fibres more quickly, causing swelling of the polyester and opening free sites and spaces in the chains of big molecules of the polyester fabrics. High temperatures at 130 °C are used in the dyeing process without the use of carriers [34]. High temperature dyeing provides advantages such as superior strength and color spread and quick dyeing time utilizing closed equipment, but it also demands a large percentage of energy consumption and may cause deformation of the cloth in some circumstances. Dispersion parameters, dye bath particle size, dye bath pH, and dyeing temperature are some of the variables that impact polyester dyeing. In polyester dyeing, dispersing agents are used to prevent dye molecules from clumping in the dye bath and to obtain an acceptable distribution of the molecules. In order to improve the solubility of the distributed dye and, consequently, the dye’s absorption on the polyester fabric, the dye bath’s particle size should be reduced. Traditional PET dyeing is conducted in a slightly acidic medium at pH values of 4.5 to 5.5. The temperature of the dyeing process has a significant impact on polyester’s dye absorption and diffusion [35]. The rate of dye absorption increases as the dyeing temperature rises because the amorphous macromolecular chains of the fibres are more mobile [36,37,38,39,40,41,42]. This review article aims to showcase our progress on the synthesis of disperse dyes derived from enaminones. The review highlights these dyes’ extra utility because of their intriguing biological action. In an effort to influence certain significant multifunctional properties, we also focused on treating polyester fabrics with nano-zinc oxide. Here, we provide an overview of our program for using enaminone-based azo dyes to explore the color depth and intensity, fastness characteristics, and antimicrobial activities of polyester fabrics dyed at 70, 80, 90, 100, and 130 °C. We also discuss how to apply zinc oxide nanoparticles for treatments of polyester fabrics in order to improve their light fastness, self-cleaning capabilities, antibacterial properties, and UV protection.

## 2. Chemistry

**Scheme 1:**
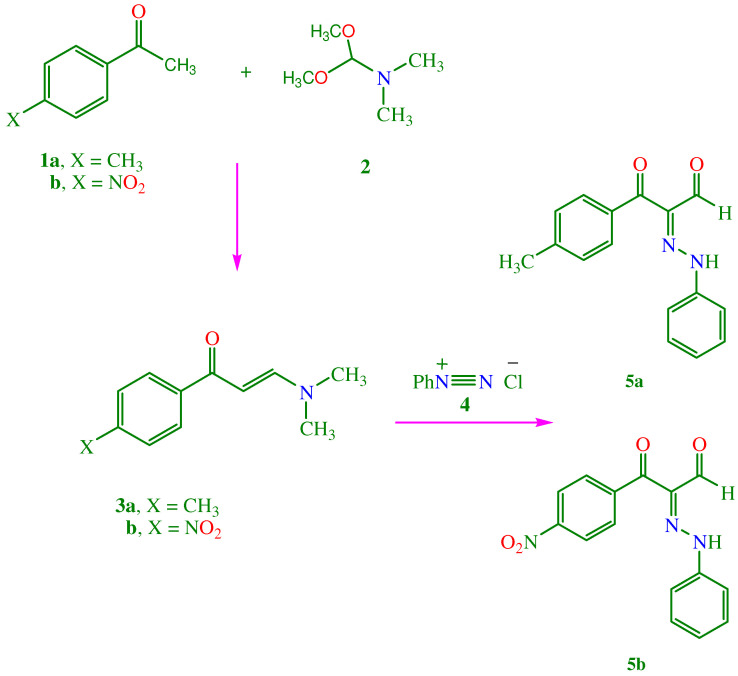
Structures of disperse dyes.

There have been reports of the synthesis of some dyes based on the 3-dimethylamino-1-p-arylpropenones moiety [33,34,35]. Here, enaminones **3a**,**b** react with phenyl diazonium salt **4** in acidic medium to produce the 3-oxo-2-(phenylhydrazono)-3-p-arylpropionaldehydes disperse colours **5a**,**b** (Scheme 1). Based on the FT IR data, the two (NH) groups of dyes, **5a** and **5b**, appeared at 3127 and 3130 cm^−1^. Also, the four carbonyl (CO) groups of dyes **5a** and **5b** appeared at 1662, 1635 cm^−1^, and 1660, 1633 cm^−1^, respectively. Based on the proton NMR data, dye **5a**’s proton NMR signal appeared at (DMSO-d_6_): δ = 14.21 (s, 1H, NH). 9.98 (s, 1H, CHO), 7.21–7.54 (m, 9H, arom-H), 2.41 (s, H, CH_3_). Consequently, dye **5b**’s proton NMR signal appeared at (DMSO-d_6_): δ = 14.18 (s, 1H, NH). 9.92 (s, 1H, CHO), 7.11–7.83 (m, 9H, arom-H).

## 3. Dyeing at Temperatures of 70, 90, and 100 °C

**Figure 1:**
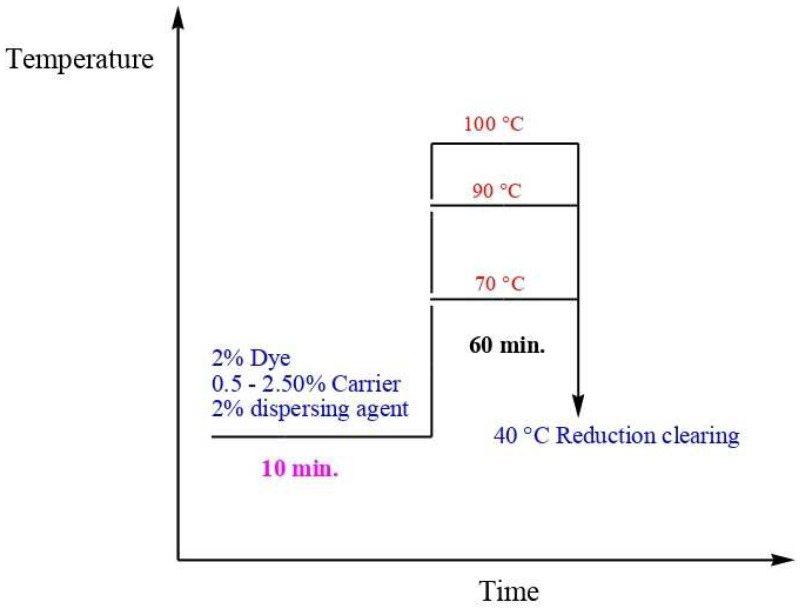
Dyeing graph at 70, 90, and 100 °C.

We dyed polyester materials in two different ways using the newly created disperse dyes after completing the synthesized process. Here, we discuss the low temperature technique once more. Next, we employed the two new disperse dyes, **5a** and **5b**, to dye polyester materials at 2% shade, either with or without using carriers (HC carrier supplied by Egyptian Turkish Co., Cairo, Egypt for auxiliaries) at different dyeing temperatures (70, 90, and 100 °C). We thus achieved a variety of color hues, from yellowish-orange to greenish-yellow (Figure 1).

It is of value to mention here that the color strength K/S and the total color difference ∆E of the dyed polyester fabrics were determined by using an UltraScan PRO D65 UV/VIS Spectrophotometer (HunterLab, Reston, VA, USA). The K/S values were determined by applying the Kubelka–Munk equation:

K/S = [(1 − R)^2^/2R] − [(1 − R_o_)^2^/2R_o_]

where R is the reflectance of colored samples and K and S are the absorption and scattering coefficients, respectively. R_o_ = decimal fraction of the reflectance of the undyed fabric. The total color difference was determined by applying the following equation:

∆E = (∆*L*^2^ + ∆*a*^2^ + ∆*b*^2^)^½^

∆E is the total color difference between the sample and the standard: (*a*) represents the red-green axis, (*L*) represents the white-black axis, and (*b*) represents the yellow-blue axis.

Dyes **5a** and **5b** demonstrated a strong affinity for the polyester materials, as indicated by the K/S values listed in Table 1.

### 3.1. Effect of Carrier Concentration 

**Figure 2:**
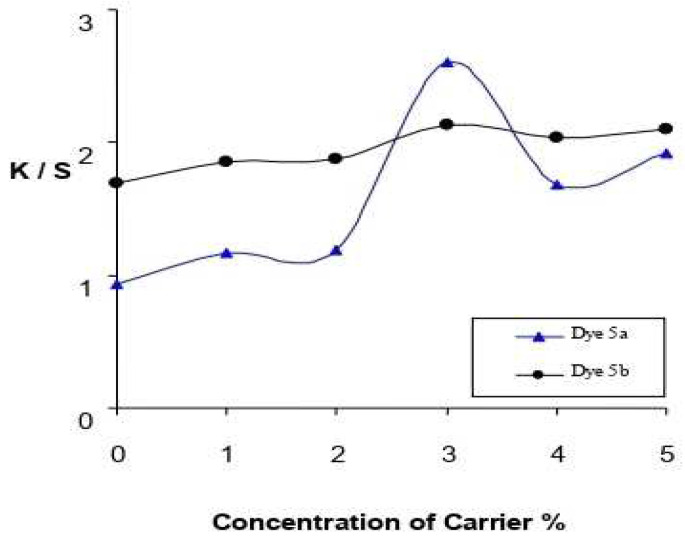
Effect of carrier concentrations on dyeing at 70 °C.

**Figure 3:**
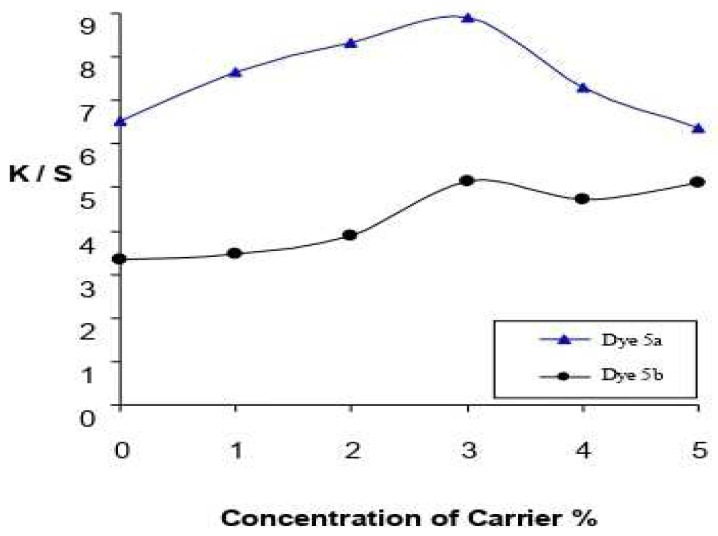
Effect of carrier concentrations on dyeing at 90 °C.

We investigated [34] the impact of carrier concentrations during low-temperature dyeing processes on polyester fabrics, namely at 70, 90, and 100 °C, when polyester fabrics are dyed with the new disperse dyes **5a** and **5b** at 70 and 90 °C. Figure 2 and Figure 3 clearly demonstrate that there is a noticeable increase in the K/S values (color strength), which reach high values at a concentration of 3% of the carrier concentration. This increase occurs when the carrier material concentration is increased from zero to 5% of the weight of the carrier material. In contrast, dye **5b**’s color intensity values K/S at 70 and 90 °C were 2.13 and 5.15, while dye **5a**’s color intensity values were 2.60 and 8.90 at those temperatures.

**Figure 4:**
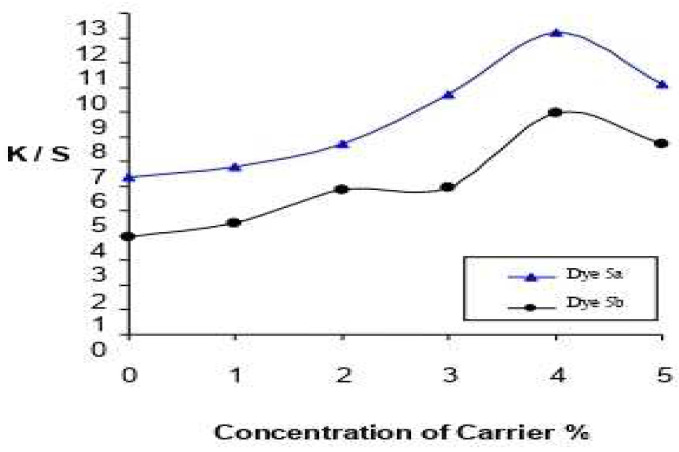
Effect of carrier concentrations on dyeing at 100 °C.

Up to a concentration of 4% of the carrier agent concentration, there is a discernible rise in the K/S values (color strength) when dying polyester materials at 100 °C with the new disperse dyes **5a** and **5b**. At 100 °C, dyes **5a** and **5b** had high color intensity values K/S of 12.21 and 8.97 (Figure 4), respectively.

According to Table 1’s results, the polyester fabric dyed with the new disperse dye **5a** has a K/S color intensity value that increases as the carrier concentration rises to 3% when dyeing at temperatures of 70 and 90 °C. This means that, in comparison to fabrics dyed without the use of carrier materials during the dyeing processes, the K/S values increase by approximately 180% and 37% compared with fabrics dyed without using the carrier during dyeing operations.

In addition, we discovered that, when dyeing polyester fabric at temperatures of 100 °C, the new disperse dye **5a** causes the color intensity value K/S to rise with an increase in the carrier concentration to 4%. This results in an approximately 93% increase in K/S values compared with fabrics dyed without using the carrier during dyeing operations. Additionally, we observed that, while dyeing at temperatures of 70 and 90 °C, the K/S color intensity value of polyester fabric colored with the novel **5b** disperse dye increases with an increase in the carrier concentration to 3%. The K/S values increased by approximately 27% and 55% compared with fabrics dyed without using the carrier during dyeing operations. Furthermore, we discovered that, when dyeing polyester fabric at 100 °C, the new dispersed dye **5b** causes the color intensity value K/S to rise with an increase in the carrier concentration to 4%. This results in an approximate 129% increase in K/S values compared with fabrics dyed without using the carrier during dyeing operations. When dyeing polyester fabrics at low temperatures [34], carrier materials are crucial because they help create more and bigger gaps in the fabric’s molecular structure where the dye molecules may disperse and spread more quickly.

### 3.2. Effect of Dyeing Temperature 

Figure 2, Figure 3 and Figure 4 show that the K/S values of the two dyes **5a** and **5b** increase as the temperature of the polyester fabric dyeing process rises, beginning at a temperature of 70 to 100 °C. First, the color strength K/S values rise by 243% and 142%, respectively, when the temperature is raised from 70 to 90 °C. Second, increasing the dyeing temperature from 90 to 100 °C results in an increase of 37% and 74%, respectively, in the K/S values of polyester fabrics dyed with dyes **5a** and **5b**.This could be because the dye molecules are present in aggregated form at low temperatures of 70 and 90°C, and as the dyeing temperature is elevated to 100 °C, the solubility of the dye molecules increases.

## 4. High Temperature Dyeing

**Figure 5:**
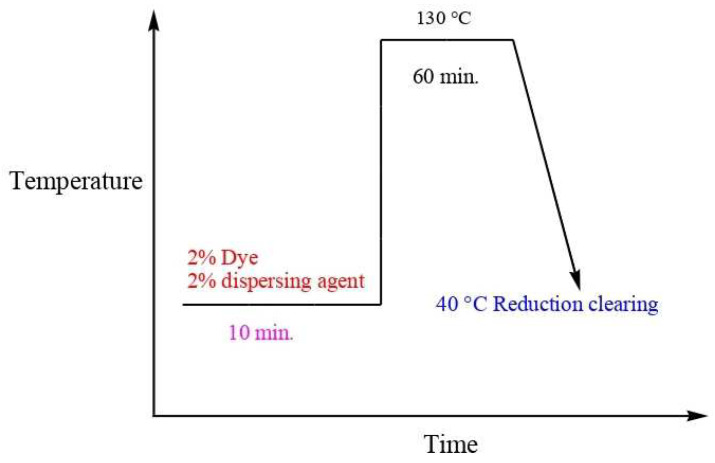
Dyeing graph at 130°C.

Additionally, we used the newly created disperse dyes **5a** and **5b** to dye polyester fabrics at a high temperature after completing the process of synthesizing them. Next, we used the two new disperse dyes, **5a** and **5b**, to dye polyester fabrics at a shade of 2% at a dyeing temperature of 130 °C (Figure 5). We thus achieved a variety of color hues, from yellowish-orange to greenish-yellow. The K/S values given in Table 2 demonstrate that all of the dyes, including **5a** and **5b**, had excellent K/S and a strong affinity for polyester textiles. Overall, we observe that dye **5a**’s color on polyester fabric has moved towards a green tint, where (a*)’s negative value, −20.37, may be found.

Furthermore, the K/S values for dyes **5a** and **5b** for polyester fabrics colored at high and low temperatures were 17.59, 16.69, 12.21, and 8.97, respectively. Table 3 presented data indicating that the color strength K/S of polyester textiles dyed at low temperatures (100 °C) was 45% and 87% lower than that of fabrics dyed at high temperatures (130 °C).

## 5. Fastness Properties

Table 4 presents the findings of our evaluation of the dyed polyester materials’ fastness against washing, rubbing, and perspiration at 100 °C. It is evident from the data that the disperse dyes exhibited outstanding fastness. On polyester materials, the dyes **5a** and **5b**’s light fastness was found to be satisfactory. It is evident to us that coloring using carrier ingredients enhances washing fastness. The findings of our evaluation of the colored polyester fabrics at 100 °C, which are shown in Table 4, unmistakably reveal that the dispersed dyes exhibited outstanding fastness to rubbing, washing, and sweating. On polyester materials, the dyes **5a** and **5b**’s light fastness was found to be satisfactory. It is evident to us that coloring using carrier ingredients enhances washing fastness.

The polyester textiles that were colored at 130 °C were assessed for stability and are included in Table 4. According to the findings, polyester fabrics treated with disperse dyes had extremely good washing fastness and great sweat and friction fastness. Ultimately, the outcomes showed a respectable level of light fastness. As a result, we treated polyester fabrics with zinc oxide nanoparticles to increase their light fastness.

## 6. Antimicrobial Activities

We discovered [33] that when testing novel synthetic disperse dyes for biological action, strong antibacterial properties were demonstrated by all of the colors examined in this investigation. The data shown in Table 5 show that while the disperse dye **5b** has activity against fungus, the disperse dye **5a** has no activity at all.

## 7. Treatment of Polyester Fabrics with ZnO NPs

In 2015 [33], we considered attempting to increase the light fastness of dyed samples and their biological activity after finding polyester materials with good light resistance but no fungal resistance. In order to add value to polyester fabrics, we treated them with nano-metallic oxides in 2016 [35], by endowing them with characteristics, enabling self-cleaning, and enhancing their resistance to UV light. We discuss these attributes in more detail below.

### 7.1. Ultraviolet Protection Factor (UPF)

**Figure 6:**
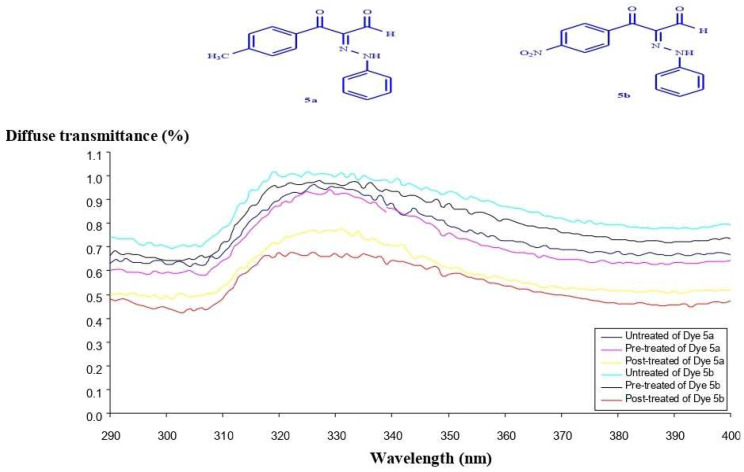
UV transmission treated polyester fabrics with ZnO NPs [35].

The Ultraviolet Protection Factor (UPF) was calculated to determine its UV protection characteristics. The UPF is a factor that endows materials with UV protection qualities. The UV protecting properties of polyester fabrics treated with ZnO nanoparticles (NPs) are displayed in Figure 6 [35]. The polyester textiles treated with ZnO NPs were 145.51 and 173.25 for dye **5a** and 131.55 and 190.59 for disperse dye **5b**, according to the UPF data in Table 6. Consequently, the UPF values provide unambiguous proof that polyester fabric is treated more effectively after dyeing. Polyester textiles go through a lot of processing before being dyed for every color that is utilized. Additionally, Table 6 shows that the UPF values for treated polyester textiles are higher than those for untreated polyester fabrics. These treated polyester fabric UPF values were as follows: dye **5a** is 141.88, and dye **5b** is 122.37 [35].

### 7.2. Self-Cleaning Evaluations

One benefit of polyester fabrics treated with metal oxide nanoparticles is their “self-cleaning” ability, which is the ability to use absorbed light to remove stains from the fabric’s surface. Research was done on the photo-degradation of coffee and methyl red stains on polyester fabrics that had been treated with nano-zinc oxide to provide them self-cleaning qualities. The photo-degradation of coffee and methyl red stains on polyester fabrics treated with nano-zinc oxide is shown in Table 7 following a 24-h UV exposure. Polyester textiles treated with zinc oxide nanoparticles showed signs of coffee staining and methyl red streaks caused by UV light. When ZnO NPs [35] are applied to polyester fabric, a thin layer of ZnO NPs forms, improving the fabric’s hydrophobic qualities. It is well known that the hydrophobic surface keeps dirt from absorbing, keeping fabric surfaces cleaner. After a full day, it was discovered that the greatest percentage of photo-degradation on the polyester fabric’s surface was between 60 and 70% for methyl red and coffee stains [35].

### 7.3. Light Fastness Evaluations 

It is worth noting that disperse azo dyes have properties and advantages such as dye strength, low cost compared to other dyes, and strong electron donating groups. On the other hand, its disadvantages are its low stability to light and low shade. As previously discussed, we treated the polyester fabrics with zinc oxide nanoparticles in an effort to enhance their light fastness feature. This process is referred to as pre-treated. On the other hand, we dyed the fabric and then gave it a treatment with zinc oxide nanoparticles, which is the so-called post-treated. Table 7 [35] presents the photo-stability measurements of all samples dyed with disperse dyes **5a** and **5b** of polyester fabrics for both pre- and post-treated with zinc oxide nanoparticles. As compared to polyester textiles that have been post-treated, the results shown in Table 7 show that treating pre-treated polyester fabrics with zinc oxide nanoparticles increases their effectiveness. Furthermore, polyester textiles dyed with disperse dyes **5a**, **5b** and treated with zinc oxide nanoparticles had a higher level of light fastness than polyester fabrics dyed with the same disperse dyes but without the treatment.

### 7.4. Antimicrobial Activity Evaluation

The biological activity of polyester fabrics dyed with disperse dyes **5a** and **5b**, both untreated and treated with zinc oxide nanoparticles, was assessed in relation to *Candida albicans* as yeast, *Bacillus subtilis*, and *Staphylococcus aureus* as Gram-positive bacteria and *Escherichia coli* and *Klebsiella pneumoniae* as Gram-negative bacteria. Table 8 indicates that the untreated and post-treated polyester fabrics dyed with disperse dyes **5a** and **5b** after the dyeing process dyed with disperse dyes **5a** and **5b**, which is called post-treatment using nano-zinc oxide, did not show antibacterial activity against all microorganisms used in this study. Polyester fabrics dyed with disperse dye **5a** and pre-treated with nano-zinc oxide show antibacterial activity against only *Bacillus subtilis* of 11 mm, while polyester fabrics dyed with disperse dye **5b** and pre-treated with nano-zinc oxide show antibacterial activity against both *Bacillus subtilis* of 8 mm and *Klebsiella pneumoniae* 10 mm. Polyester fabrics dyed with disperse dyes **5a** and **5b** and pretreated with nano-zinc oxide did not show antibacterial activity against *Staphylococcus aureus*, *Escherichia coli*, and *Candida albicans* [35].

The mechanism for Nano ZnO’s well-known antibacterial activity could be that it aids in the formation of peroxide, which itself has antibacterial properties, or it could act through ZnO nanoparticles, which could disrupt bacterial membranes and inhibit their growth.

## 8. Conclusions

The novel disperse dyes 3-Oxo-2-(phenylhydrazono)-3-p-tolyl-propionaldehyde (**5a**) and 3-(4-Nitrophenyl)-3-oxo-2-(phenylhydrazono)-propionaldehyde (**5b**) were demonstrated to be obtained in this review.3-Dimethylamino-1-arylpropenones are combined with phenyl diazonium salt to achieve this. Utilizing both two temperature dyeing techniques at 100 and 130 °C, the novel disperse dyes were employed to dye polyester fabrics. Consequently, we obtained polyester fabrics with greenish-yellow and orange hues, which possess good light fastness, superior washing, rubbing, and perspiration fastness. The synthesized dispersing dyes’ antibacterial efficacy against yeast, fungus, and Gram-positive and Gram-negative bacteria is demonstrated and discussed. ZnO NPs were applied to the newly dyed polyester fabrics using disperse dye in order to improve their light fastness, antibacterial resistance, and self-cleaning qualities. Next, the ultraviolet protection factor data are shown, showing that polyester textiles treated with ZnO NPs have UV protecting properties of 145.51 and 173.25 for disperse dye **5a** and 131.55 and 190.59 for disperse dye **5b**. The photo-catalytic activity of coffee and methyl red stains were used to assess the self-cleaning capability. Lastly, the light fastness and antibacterial capabilities of polyester textiles treated with ZnO NPs were demonstrated. Additionally, the added benefit of using ZnO NPs to give polyester fabrics significant properties that they lacked previously was explored.

## Figures and Tables

**Table 1:** Optical measurements of dyes **5a**, **b** [34].

* Dyeing Temperature	Dye 5a		Dye 5b		% Carrier
	∆E	K/S	∆E	K/S	
70 °C	87.98	0.93	23.29	1.69	0
	88.40	1.17	28.54	1.85	1
	88.19	1.19	19.33	1.87	2
	88.40	2.60	32.78	2.13	3
	89.32	1.68	31.13	2.04	4
	90.19	1.92	33.97	2.10	5
90 °C	94.56	6.53	46.00	3.34	0
	95.60	7.65	47.55	3.47	1
	96.36	8.32	48.99	3.90	2
	97.14	8.90	50.10	5.15	3
	95.10	7.30	48.29	4.73	4
	94.54	6.37	50.40	5.12	5
100 °C	94.07	6.34	48.20	3.93	0
	95.16	6.80	51.11	4.49	1
	96.31	7.72	56.34	5.85	2
	98.63	9.70	52.75	5.92	3
	99.58	12.21	58.04	8.97	4
	98.44	10.12	57.21	7.71	5

* Dyeing process: 2% shade dye dispersion was introduced dropwise while stirring to a 50:1 liquor-to-dye bath that contained a Matexil DA-N as dispersing agent. The polyester textiles were added when the dye bath’s pH was brought to 4.50 by acetic acid. The dyeing process lasted 60 min at 100 °C. Following the dyeing process, the materials underwent a thorough washing, surface reduction cleaning (2 g NaOH + 2 g sodium hydrosulphite)/L), and 2% nonionic detergent soaping. The samples were heated to 80 °C for 45 min in this solution. After thoroughly rinsing with cold water and neutralizing with 1 g/L acetic acid for 5 minutes at 40 °C, the dyed samples were taken out and dried with tap water.

**Table 2:** Optical measurements of dyed polyester fabrics at 130 °C.

Dye No.	Color Shade on Polyester	L*	a*	b*	K/S	Reference
**5a**	Greenish-yellow	77.22	−20.37	53.94	17.59	[33]
**5b**	Yellowish-orange	67.58	9.29	61.34	16.69	

Dyeing: 2% shade dye dispersion was introduced dropwise while stirring to a 50:1 liquor-to-dye bath that contained a Matexil DA-N as dispersing agent. The polyester textiles were added when the dye bath’s pH was brought to 4.50 by acetic acid. The dyeing process lasted 60 min at 130 °C. Following the dyeing process, the materials underwent a thorough washing, surface reduction cleaning (2 g NaOH + 2 g sodium hydrosulphite)/L), and 2% nonionic detergent soaping. The samples were heated to 80 °C for 45 min in this solution. After thoroughly rinsing with cold water and neutralizing with 1 g/L acetic acid for 5 minutes at 40 °C, the dyed samples were taken out and dried with tap water.

**Table 3:** Color strengths of dyes **5a** and **5b**.

Dye No.	L*	a*	b*	K/S	Reference
Dyeing at 100 °C					
**5a**	84.81	−14.26	52.16	12.21	[34]
**5b**	78.08	−0.21	51.20	8.97	
Dyeing at 130 °C					
**5a**	77.22	−20.37	53.94	17.59	[33]
**5b**	67.58	9.29	61.34	16.69	

**Table 4:** Fastness properties of polyesters fabrics.

Dye No.	Washing Fastness			Light Fastness	Rubbing Fastness		Perspiration Fastness					
							Alkaline			Acidic		
	Alt	SC	SW		Dry	Wet	Alt	SC	SW	Alt	SC	SW
Dyeing at 100 °C												
**5a**	5	5	5	3–4	5	5	5	5	5	5	5	5
**5b**	5	5	5	3–4	5	5	5	5	5	5	5	5
Dyeing at 130 °C												
**5a**	4–5	4–5	3–4	3	5	5	5	5	5	5	5	5
**5b**	4–5	4–5	4	3–4	5	5	5	5	5	5	5	5

**Table 5:** Diameter of the zones of inhibition of the tested dyes.

Dye No.	* Inhibition Zone Diameter (Nearest mm)						Reference
	G-Ve Bacteria		Yeast	Fungi	G+Ve Bacteria		
	*E. coli*	*P. aeruginosa*	*C. albicans*	*A. Niger*	*B. cereus*	*S. aureus*	
**5a**	9	8	NI	NI	11	11	[33]
**5b**	10	14	20	24	18	12	

NI: No inhibition zone. * Antimicrobial Activities Test: Using the Agar-well diffusion technique, the antibacterial properties of dispersed dyes were evaluated against six distinct microbial cultures. The test included pure cultures of *Aspergillum niger* (fungus), *Candida albicans* (yeast), *Escherichia coli* and *Pseudomonas aeruginosa* (G-bacteria), and *Bacillus cereus* and *Staphylococcus auerus* (G+bacteria). Whereas 0.1 mL of the yeast and fungus were placed on potato dextrose agar (PDA), an aliquot of 0.1 mL of each bacterial strain was inoculated and spread on nutrient agar (NA). Each tested dye was added to the inoculated plates in 100µL increments, resulting in a final concentration of 100 mg mL^−1^. The sterile cork borer was used to create 6 mm wells, which held the dyes. The NA plates were incubated for 24 h at 37 °C, and the PDA plates were incubated for 24 to 48 h at 25 °C. The well-adjacent zones of inhibition were identified. Diameter of well: 6 mm.

**Table 6:** UPF of treated polyester fabrics with ZnONPs.

Dye No.	Treatment	UPF	ZnO %	Reference
	Blank	19.420	0	[35]
**5a**	Untreated	141.88	0	
	Pre-treated	145.51	2.5	
	Post-treated	173.25	2.0	
**5b**	Untreated	122.37	0	
	Pre-treated	131.55	2.5	
	Post-treated	190.59	2.5	

**Table 7:** Methyl red stain, coffee stain, and light fastness evaluation.

Dye No.	Treatment	ZnO%	Methyl Red Stain		Coffee Stain		Light Fastness
			∆E	K/S	∆E	K/S	
		untreated	67.23	5.63	66.77	5.98	3–4
**5a**	Pre-treated	0.5	61.21	4.36	57.41	3.45	5
		1.0	53.41	2.84	52.1	2.78	2–3
		1.5	53.41	2.73	57.45	3.45	2–3
		2.0	51.04	2.37	54.85	2.82	3–4
		2.5	53.32	2.72	58.71	3.61	3
	Post-treated	0.5	60.20	4.03	54.16	2.88	3
		1.0	59.41	3.84	52.67	2.66	3
		1.5	60.88	4.08	53.09	2.86	3
		2.0	55.44	3.23	53.12	2.78	3
		2.5	60.36	3.92	63.47	4.99	3
		untreated	50.02	3.63	51.92	3.99	4
**5b**	Pre-treated	0.5	52.62	4.15	54.24	4.27	5
		1.0	54.36	4.37	58.72	5.05	5
		1.5	53.29	4.27	54.85	4.31	5
		2.0	52.76	3.76	54.82	4.19	5
		2.5	56.23	3.93	60.71	4.79	4–5
	Post-treated	0.5	54.42	4.31	51.02	3.96	5
		1.0	55.32	4.28	51.76	3.90	5
		1.5	54.37	4.43	50.11	4.24	4
		2.0	53.73	4.12	50.21	3.95	4–5
		2.5	60.18	5.37	56.47	4.88	4–5

Pre-treatment: The fabric samples were soaked in a 10 g/L nonionic detergent solution for 10 min, and then they were gently stirred for 15 min to disperse ZnO NPs (0–2.5% *w*/*w*). After the materials were compressed to eliminate extra dispersion, they were baked for 10 minutes at 70 °C. For three minutes, the fabrics were queried at 140 °C. The fabrics were cleaned for 15 min at 60 °C in an aqueous solution with a liquor ratio of 1:50 and 3 g/L nonionic detergent solution. Post-treatment: Following dyeing, the fabric samples were gently stirred for 15 min while being immersed for 10 min in a 10 g/L nonionic detergent solution containing ZnO NPs (0–2.5% *w*/*w*). After the materials were compressed to get rid of extra dispersion, they were baked for 10 minutes at 70 °C. For three minutes, the fabrics were queried at 140 °C. The fabrics were cleaned for 15 min at 60 °C in an aqueous solution with a liquor ratio of 1:50 and 3 g/L nonionic detergent solution. Photo-induced discoloration onto polyester fabric: Spots of 60 µL coffee or 2.5 g/10 mL of methyl red were applied to polyester fabric that had not been treated with ZnO. The samples were exposed to UV light for a full day.

**Table 8:** Inhibition zone diameters of the dyed polyester fabrics [35].

Dye No.	ZnO% & Treatment	Inhibition Zone Diameter (Nearest mm)					Reference
		G^+^Inhibition Zone		Yeast	G^-^Inhibition Zone		
		*Bacillus subtilis*	*Staphylococcus aureus*	*Candida albicans*	*Escherichia coli*	*Klebsiellapneumoniae*	
**5a**	Untreated	NI	NI	NI	NI	NI	[35]
	2.5 Pre-treatment	11	NI	NI	NI	NI	
	2.5 Post-treatment	NI	NI	NI	NI	NI	
**5b**	Untreated	NI	NI	NI	NI	NI	
	2.5 Pre-treatment	8	NI	NI	NI	10	
	2.5 Post-treatment	NI	NI	NI	NI	NI	

NI: No inhibation zone. Antimicrobial Activities Test: We assessed how well dyed polyester fabrics inhibited the growth of *Bacillus subtilis*, *Candida albicans*, *Staphylococcus sureus*, and *Klebsiella pneumoniae*. It is important to note that the microorganisms used in this investigation, *Eschercia coli*, were supplied by the Soil Microbiology Unit of the Desert Research Centre in Cairo, Egypt. Filter paper discs were used in agar diffusion studies to test antimicrobial activity.

## Data Availability

Not applicable.
